# Ionic Liquids-Polymer of Intrinsic Microporosity (PIMs) Blend Membranes for CO_2_ Separation

**DOI:** 10.3390/membranes12121262

**Published:** 2022-12-13

**Authors:** Giuseppe Ferraro, Carmela Astorino, Mattia Bartoli, Alberto Martis, Stefania Lettieri, Candido Fabrizio Pirri, Sergio Bocchini

**Affiliations:** 1 Center for Sustainable Future Technologies (CSFT), Istituto Italiano di Tecnologia (IIT), Via Livorno 60, 10144 Turin, Italy; 2 Dipartimento di Chimica Generale ed Organica Applicata, Università di Torino, Corso Massimo D’Azeglio 48, 10125 Turin, Italy; 3 Department of Applied Science and Technology, Politecnico di Torino, Corso Duca Degli Abruzzi 24, 10129 Turin, Italy

**Keywords:** ionic liquids, polymers of intrinsic microporosity, CO_2_, gas separation, supported ionic liquids membranes, carbon capture and storage, gas solubility, flow cell

## Abstract

Membranes with high CO_2_ solubility are essential for developing a separation technology with low carbon footprint. To this end, physical blend membranes of [BMIM][Ac] and [BMIM][Succ] as Ionic Liquids (ILs) and PIM-1 as the polymer were prepared trying to combine the high permeability properties of PIM-1 with the high CO_2_ solubility of the chosen ILs. Membranes with a PIM-1/[BMIM][Ac] 4/1 ratio nearly double their CO_2_ solubility at 0.8 bar (0.86 cm^3^ (STP)/cm^3^ cmHg), while other ratios still maintain similar solubilities to PIM-1 (0.47 cm^3^ (STP)/cm^3^ cmHg). Moreover, CO_2_ permeability of PIM-1/[BMIM][Ac] blended membranes were between 1050 and 2090 Barrer for 2/1 and 10/1 ratio, lower than PIM-1 membrane, but still highly permeable. The here presented self-standing and mechanically resistant blend membranes have yet a lower permeability compared to PIM-1 yet an improved CO_2_ solubility, which eventually will translate in higher CO_2_/N_2_ selectivity. These promising preliminary results will allow us to select and optimize the best performing PIM-1/ILs blends to develop outstanding membranes for an improved gas separation technology.

## 1. Introduction

To decrease the anthropogenic climate change due to global greenhouse gas (GHG) emissions [1], which is mostly caused by carbon dioxide (CO_2_) [2], and to meet the long-term goals of the Paris Agreement (Adoption of the Paris Agreement FCCC/CP/2015/L.9/Rev.1 (UNFCCC, 2015) [3], one of the most promising carbon mitigation strategies is the Carbon Capture and Storage (CCS) [4] being an effective method for the recovery of CO_2_ from power plant flue gas (CO_2_/N_2_) [5], syngas in hydrogen production (CO_2_/H_2_), natural gas, and biogas (CO_2_/CH_4_) [6]. Membranes have been recently identified as one of the most promising and emerging technologies for the separation processes in carbon sequestration, as they are cost-effective and not require thermal driving force during their use, reducing the energy needs of 90% compared to the cryogenic distillation process which is an energy-intensive separation method (10–15% of total energy consumption) [6]. Therefore, the development of membranes with high CO_2_ solubility is essential for developing a separation technology with a low carbon footprint.

Selectivity (S) and permeability (P) of the membrane are the main factors that oversee the efficiency of the membrane in gas separation technology. The selectivity is the ability of the membrane to physically, chemically, or by pore size exclusion, separate one species from a mixture, while the permeability is affected by pore size and other surface characteristics of the membranes. The microporosity of the polymer can be finely tuned to enhance their gas diffusion and thus permeability. An example was proposed by Budd and McKeown in 2004 [7] by reporting on a polymer of intrinsic microporosity (PIM) named PIM-1, which microporosity (pore size < 2 nm) is created simply because the highly rigid and contorted molecular structure cannot fill space efficiently when packed. PIMs create self-standing membranes with high surface area (600–900 m^2^ g^−1^). In general, all polymers contain some void space or free volume, however, as in the case of PIM, the voids need to be interconnected, enabling the free movement of gas molecules, generating high gas adsorption.

Membranes prepared from PIM-1 show a large area of free volume producing high permeability for many gases [8], especially for CO_2_ (4000 to 10,000 Barrer) being highly soluble in this polymer [5]. Therefore, PIMs have been largely used in gas separation technology, as they are able to reach high values not only in terms of permeability, but also in selectivity for O_2_/N_2_, CO_2_/CH_4_ and CO_2_/N_2_ [9,10]. PIMs are also polymers that are easy to synthesize, which makes them great candidates for industrial scale-up. However, there is a trade-off between selectivity and permeability, meaning that polymers with higher permeability will generally be less selective and vice versa, as demonstrated by Robeson [11,12]. This is a massive drawback hardly overcoming. This happen mainly because the solubility selectivity is modest. Thus, the improved performance of PIMs over Robeson’s upper bound is linked to the increase of solubilities while not decreasing the permeability [8].

One of the strategies to obtain polymer-based composite materials with improved gas transport properties beyond the Robeson’s upper bound is to combine polymers with fillers (being solid or liquids), resulting in a synergy being able to positively enhance their properties. Although the physical blending approach can have some drawback, such as miscibility and homogeneity of the blend components, it is still one of the most cost effective and fast alternatives to enhance the physical properties of pristine PIMs polymers [13,14,15,16,17]. In particular, an attracting material highly soluble to CO_2_ and largely studied for CCS [18], are ionic liquids (ILs). ILs have also attracted attentions as a green solvent compared to traditional ones, besides being chemically and thermally stable, having a negligible vapor pressure and for their ease and scalable synthesis [19,20]. ILs could absorb CO_2_ either physically or chemically [20]. Chemical sorption is always favorite at lower pressure and thus effective ILs that can interact chemically with CO_2_ were chosen. One of the best ILs for CO_2_ absorption are imidazolium based ionic liquids having great CO_2_ absorption properties because the CO_2_ is believed to form a complex with the acidic C-2 position in the imidazolium ring [21]. The 1-Butyl-3-methylimidazolium (BMIM) cation, due to its longer side chain, is expected to better interact with polymer matrix and thus decrease leaching of the IL during operation. Therefore, 1-Butyl-3-methylimidazolium acetate [BMIM][Ac] and 1-Butyl-3-methylimidazolium succinimidate [BMIM][Succ] were selected as ILs. In particular, succinimidate as an anion is theoretically able to absorb a high amount of CO_2_ (>1 mol CO_2_/mol IL). We estimate by preliminary absorption test on pure ILs [BMIM][Ac] and [BMIM][Succ] a CO_2_ uptake of 7.6 wt% and 10.9 wt%, respectively. However, it is worth highlighting that the capture in [BMIM][Succ], according to literature, should be at least equimolar, however the molar efficiency considerably lower than 1. Most probably, the high viscosity of [BMIM][Succ] hindered gas diffusion and equilibrium condition was not achieved, despite the high solubility of CO_2_ of IL.

The aim of this work was to prepare physical blend membranes of [BMIM][Ac] and [BMIM][Succ] as ILs, and PIM-1 as the polymer, to combine the high permeability properties of PIM-1 with the high CO_2_ solubility of the chosen ILs. These preliminary results will allow the selection of the best performing blended membrane for a future investigation on the effect of ILs on the gas selectivity (CO_2_/N_2_) of the blends. We expected the novel membranes to have a lower permeability compared to PIM-1 alone membranes, in trade of an improved CO_2_ solubility and eventually improved CO_2_/N_2_ selectivity.

## 2. Materials and Methods

### 2.1. Chemicals

All solvents and reagents were used as received without further purification. 2,3,5,6-Tetrafluoroterephtalonitrile were purchased from FluoroChem (Hadfield, UK). ILs [BMIM][Ac] and [BMIM][Succ] were received from Iolitec (Heilbronn, Germany). All the other reagents and solvents were purchased from Merck-Sigma-Aldrich (St. Louis, MO, USA).

### 2.2. Synthesis of PIM-1

**Figure 1:**
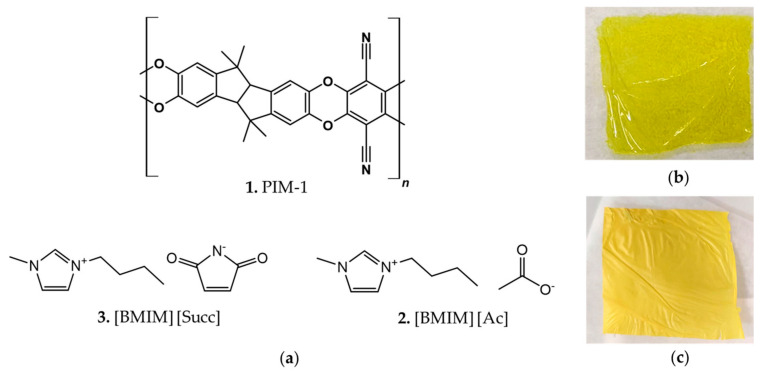
(**a**) Molecular structures of PIM-1(**1**), 1-Butyl-3-methylimidazolium succinimidate [BMIM][Succ] (**2**) and 1 -Butyl-3-methylimidazolium acetate [BMIM][Ac] (**3**); (**b**) Self-standing PIM-1 membrane; (**c**) Self-standing PIM-1/[BMIM][Succ] 2/1 membrane.

PIM-1 (Figure 1a) was synthetized following a previously reported procedure [7]. Briefly, a mixture of anhydrous K_2_CO_3_ (5.53 g, 0.04 mol, FW = 138.205), 3,3,3′,3′-Tetramethyl-1,1′-spirobiindane-5,5′,6,6′-tetraol (3 g, 0.008813 mol, FW = 340.41), and 2,3,5,6-Tetrafluoroterephthalonitrile (1.76 g, 0.008813 mol, FW = 200,096) in dry DMF (60 mL) was stirred at 65 °C for 72 h under N_2_ atmosphere. The crude was then poured in 400 mL of DI water and the precipitated washed by centrifugation several times in water and methanol. After drying under vacuum overnight a bright yellow solid was obtained in a quantitative yield.

### 2.3. Membrane Preparation

PIM-1/ILs membranes were prepared by a solvent casting method. PIM-1 was first dissolved in chloroform and stirred for 15 min at 40 °C. Subsequently, IL was added in the solution and stirred for 15 min at 40 °C. PIM-1 and ILs films were prepared with a total 2 wt% polymer concentration in chloroform. The blend ratio PIM-1/IL contents varies from 10/1, 4/1, and 2/1 where IL is [BMIM][Ac] or [BMIM][Succ] (Figure 1a). The blended solution was cast onto a leveled nylon substrate at ambient temperature. The polymer films were formed after the evaporation of the solvent (Figure 1b,c). The resultant membranes were dried at 40 °C under vacuum for at least 16 h to remove the remained traces of solvent. The membranes were labeled as “PIM-1/IL ratio composition”, for example, PIM-1/[BMIM][Ac] 10/1. The thicknesses of the casted films were around 1 µm, while the densities were between 0.979 and 1.955 (g/cm^3^) (data reported in the Supplementary Materials, Table S1).

### 2.4. Physicochemical Characterization of Membranes

Thermal degradation analysis (TGA) was performed using a thermogravimetric analyzer NETZSCH TG 209 F3 Tarsus (Selb, Germany). An amount of 10–15 mg of the sample was burned in an alumina pan under airflow (20 mL min^−1^) with N_2_ as a protective gas (20 mL min^−1^) in a temperature range of 30–800 °C (heating rate 10 °C min^−1^). The TGA spectra and degradation weight losses are reported in the Supplementary Materials (Figure S1, Table S2). Attenuated total reflection infrared spectroscopy (ATR-IR) was employed to characterize the membranes. Measurements were carried out on a Bruker Tensor II Fourier transform spectrophotometer (Billerica, MA, USA). The spectra were acquired by accumulating 64 scans (64 for the background spectrum) in 4000–600 cm^−1^ range with a resolution of 2 cm^−1^. The Tg was defined as the midpoint of the heat capacity change observed in the DSC thermogram. FESEM measurements were performed on a Zeiss SupraTM 25 (Oberkochen, Germany). Pure CO_2_ absorption-desorption measurements were performed using a Surface Measurement System, Dynamic Vapor Sorption (DVS) instrument. The pure CO_2_ isotherms were carried out at a constant temperature of 40 °C while increasing the pressure up to 0.8 bar (20% of p/p_0_ increase at each step). The equilibrium criterion condition for each step was chosen as dm/dt = 0.001% min^−1^. Gas chromatograph (µGC, Inficon Fusion^®^; Bad Ragaz, Switzerland), equipped with two columns (a 10 m Rt-Molsieve 5A and an 8 m Rt-Q-Bond) and micro thermal conductivity detectors Zeiss SupraTM 25 (Oberkochen, Germany). The thickness of the membranes was measured using a Digimatic indicator (IDC-112B-5) (Mitutoyo, Kawasaki, Japan) with an accuracy of 1 µm. The thickness of the membranes recorded is an average value obtained from at least 25 different points of the membrane.

### 2.5. Solubility Measurements

Gas permeation characteristics of PIM-1 based blends were studies, especially its selectivity was evaluated being, with the permeability, one of the main factors that indicate their overall efficiency in gas separation technology. The measurements were performed using a Dynamic Vapor Sorption (DVS) instrument following the method previously described [22]. If the diffusion process obeys Fick’s law and the down-stream pressure is much less than the up-stream pressure, the permeability P of the membranes, expressed in Barrer (10^−10^ cm^3^ (STP) cm/(cm^2^ s cmHg)) [23], is given by (1)

P_A_ = D_A_ × S_A_, (1)

where D_A_ is the average diffusion coefficient, and S_A_ is the solubility of penetrant A in the polymer. Solubility measurements were performed at for four partial gas pressure (0.2, 0.4, 0.6, 0.8 Bar) at 40 °C. The experiments were performed at least two times for each sample at the different pressure values.

### 2.6. Permeability Measurement

**Figure 2:**
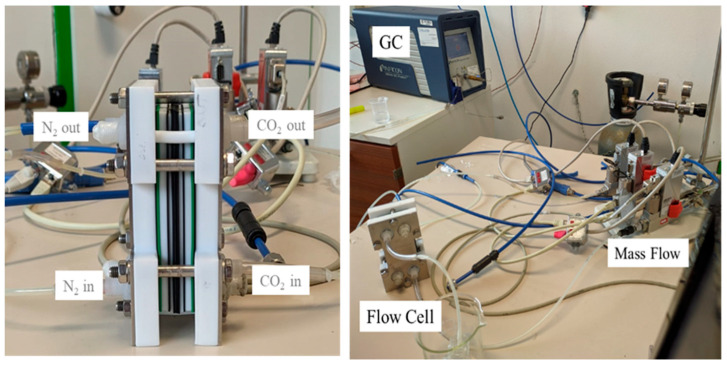
Experimental set up consisting of gas chromatograph, cell, and mass flow.

The experimental set-up used is shown in Figure 2. The flow cell consists of two gas chambers separated by the membrane the exposed area is 10 cm^2^. The chambers were kept at 25 °C and at 1 absolute bar. In the first chamber 20 mL min^−1^ CO_2_ was fluxed, while in the second chamber, a flux of 50 mL min^−1^ was used to convey CO_2_ to the gas chromatograph (µGC, Inficon Fusion^®^), equipped with two columns (a 10 m Rt-Molsieve 5A and an 8 m Rt-Q-Bond). Micro thermal conductivity detectors monitored the signal of N_2_ and CO_2_. The permeability was calculated through the evaluation of CO_2_ flux from first chamber to the second chamber divided by membrane surface and multiplied by membrane thickness. The experiments were performed at least two times for each sample at the different pressure values. The results were reported in Barrer (Section 3.3).

## 3. Results and Discussion

### 3.1. Physico-Chemical Properties

#### 3.1.1. Attenuated Total Reflectance (ATR)

**Figure 3:**
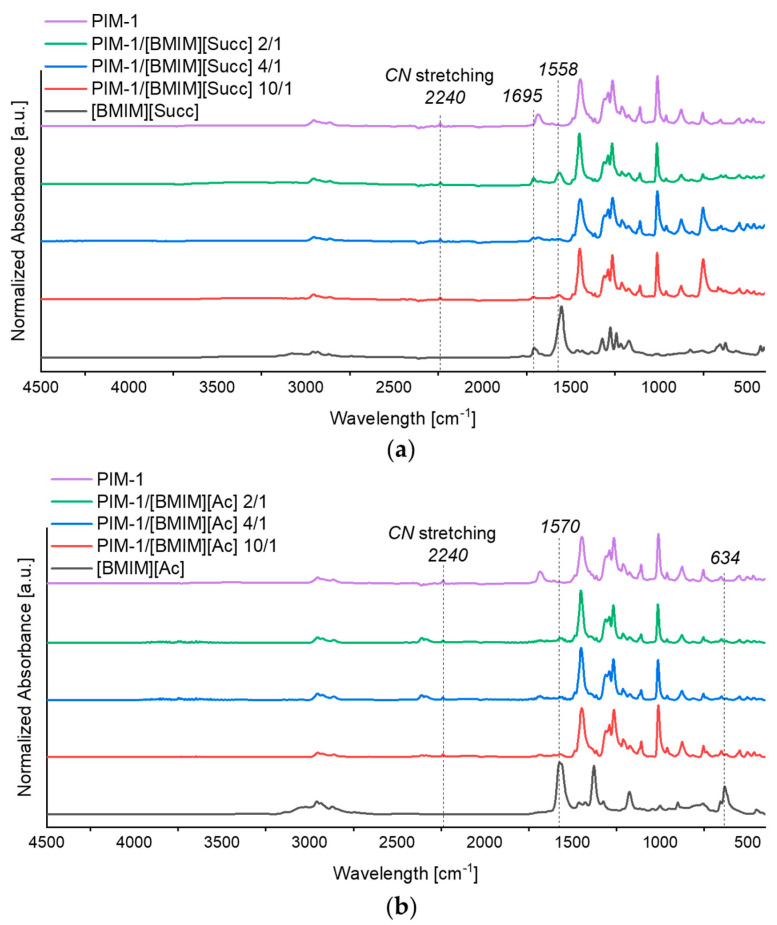
Attenuated total reflectance (ATR) spectra of blended PIM-1/ILs membranes compared to PIM-1: (**a**) [BMIM][Succ] spectra; (**b**) [BMIM][Ac] spectra.

ATR was performed on blended membranes and bare PIM-1 membrane to prove the successful introduction of ILs within the PIM-1 -based films. All spectra are shown in Figure 3. In the PIM-1 membrane spectrum, the typical peaks of the polymer are present. In particular, the C-O-C stretching peak of the aromatic ethers and the CN stretching of the nitrile group at 2240 cm^−1^ are present [24]. The imidazolium in-plane ring modes present in both ILs are located at (∼1560 cm^−1^) for [BMIM][Ac] and [BMIM][Succ] [25]. In Figure 3a, the presence of [BMIM][Succ] in the membrane is confirmed from the presence of the peak at 1558 cm^−1^ referring to the carbonyl stretching peak of succinimidate anion of [BMIM][Succ]. The appearances of characteristic bands for [SUC]-anion at 1695 cm^−1^ and 1064 cm^−1^ are attributed to C=O and C–N [26]. As expected, the increase of the typical ILs peaks intensities is directly linked with the increase of ILs % in the blend.

The imidazolium vibration partially superimposes with the asymmetric stretching (1570 cm^−1^) of the ATR spectra reported in Figure 3b of the acetate anion of [BMIM][Ac] [27]. The acetate bending is instead at 634 cm^−1^. The presence of both absorption in PIM-1/[BMIM][Ac] blends are shown, confirming the successful incorporation ILs in PIM-1 polymer.

#### 3.1.2. FESEM

**Figure 4:**
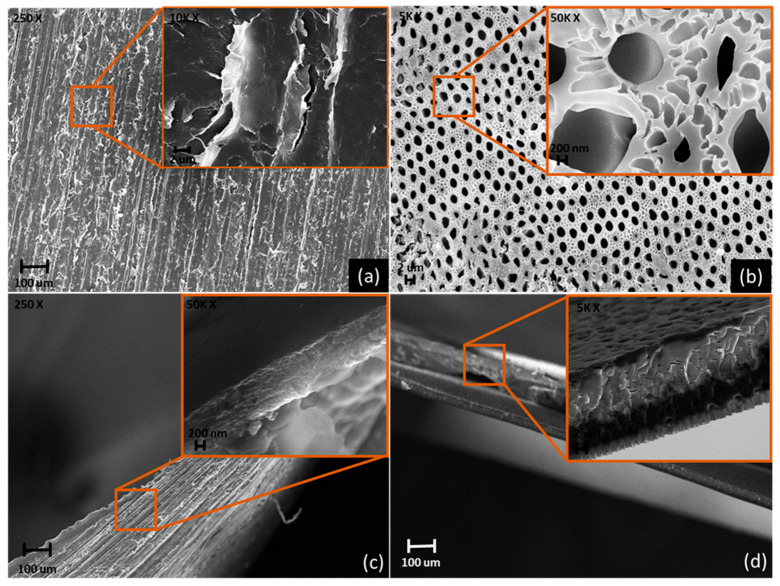
FESEM capture of surface (**a**) PIM-1, (**b**) PIM-1/[BMIM][Ac] 10/1 and their cross sections (**c**,**d**), respectively.

In Figure 4, the FESEM capture of representative of membrane with and without the addition of ILs. PIM-1 showed a smooth surface without appreciable porous network with cracks due to the intrinsically brittleness of the polymeric film as shown in Figure 4a highlight. As reported in Figure 4c, PIM-1 cross section shows a thick ranging from 13 up to 20 µm confirming the absence of diffuse pores. The PIM-1/ILs showed a very different topology characterized by porous with an average diameter size of up to 1.5-µm (Figure 4b). Furthermore, the cross section for the sample shown in Figure 4d shows the presence of small channels inside the film bulk and average thickness comparable with neat PIM-1. The FESEM analysis clearly enlightens the porous formation as a consequence of the addition of ILs without altering the thickness of the specimens. Additional FESEM capture of the here presented membranes are reported in the Supplementary Materials Figure S2.

### 3.2. Pure CO_2_ Absorption

The solubility of CO_2_ in PIM-1 is usually quite high due to the nitrile groups that may be assumed to enhance both the strength of intermolecular forces and, because of their lateral position, the free volume, giving rise to even higher apparent solubilities. The high apparent solubility of gases in PIMs thus it is attributed mainly to their microporous character, which provides a high capacity for gas [10].

**Figure 5:**
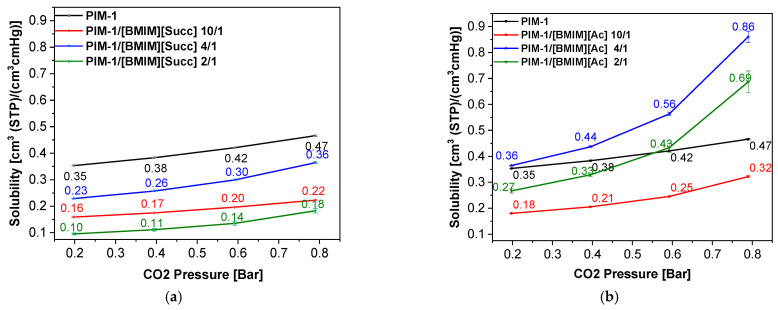
CO_2_ solubility of blended membranes at four different CO_2_ pressure of 0.2, 0.4, 0.6, 0.8 bar of [BMIM][Succ] (**a**) and [BMIM][Ac] (**b**).

Once [BMIM][Succ] is introduced, the CO_2_ solubility in the blended membranes decrease in a first instance to the following order: 0 > 4/1 > 10/1 > 2/1 (Figure 5a, Table 1). 

Again, the blend with the 4/1 of IL has the higher solubility compared to the other blended membranes; we speculate that there is a trade-off between the beneficial effect of ILs in enhancing the solubility and their “negative” limiting the free volume. Indeed, the ILs are filling the empty voids of the PIM-1, thus a decreased diffusion of gas molecules is expected. The reason of this decrease is due to the change in the mechanism that the gas molecules have undergone when passing through a membrane in the presence or absence of the ILs. In fact, the gas molecules mechanism changes from a molecular sieving mechanism [28] to an adsorption and diffusion into a liquid phase one when the ILs is added. Moreover, the high viscosity of [BMIM][Succ] could further limit the diffusion of CO_2_ in the membrane. Thus, despite the high solubility of CO_2_ in [BMIM][Succ] that could reach an uptake of 10.9 wt% at CO_2_ pressure of 1 bar, the inclusion of this ILs into the membrane decreases the total solubility (Figure 5a, Table 1).

On the contrary, the solubility coefficient of CO_2_ is found to increase significantly with the increment of [BMIM][Ac] from 10/1 to 2/1, compared to pure PIM-1 membrane, with the higher increase in solubility for PIM-1/[BMIM][Ac] 4/1 reaching and surpassing the PIM-1 for high pressures (Figure 5b, Table 1). This result was expected since [BMIM][Ac] has a high solubility specifically for CO_2_ [29].

### 3.3. Flow Cell Measurements

The permeability is given from the product of diffusion coefficient for the solubility (Section 2.5. Solubility measurements). Diffusion is expected to decrease with the increase of ILs content being diffusion into the empty microporosity much easier than in the presence of a liquid. In fact, the newly synthesized PIM-1/ILs show lower CO_2_ permeability coefficients for all blends compared to the pure PIM-1. The addition of [BMIM][Succ] onto PIM-1 generates a decrease in permeability an order of magnitude lower compared to PIM-1 (Table 2), where the permeability increase proportionally to the ILs content. 

As the CO_2_ solubility also plays a role in the permeability values, it is not surprising that the introduction of [BMIM][Succ] into the polymer plays a general “negative” effect, as the solubilities of the membranes containing this ILs are lower than PIM-1 (Table 1). Instead, the overall “negative” effect on the permeability coefficient of [BMIM][Ac] is limited, as the high CO_2_ solubility (Table 1) of these blends positively contribute to create membranes with great permeability. The optimization of this membrane will need to consider the fine equilibrium between gas diffusion, which is affected by the presence of ILs into the empty voids of PIMs, and the CO_2_ solubility, which can be enhanced by increasing the amount of ILs. However, there is a limit in the amount of ILs that can be introduced into the blends, as a loss of mechanical properties or ILs bleaching can be envisage.

For comparison, Table 3 shows CO_2_ permeability values of other PIM-1/ILs membranes published till now. Compared with relevant literature data, the PIM-1/ILs membranes developed in this work show great if not better CO_2_ permeability.

## 4. Conclusions

Self-standing and mechanically resistant blend membranes of the ILs [BMIM][Succ] and [BMIM][Ac] and a polymer of intrinsic microporosity (PIM-1) were successfully prepared. The ILs were chosen for their high CO_2_ solubility, while PIM-1 was chosen for its high gas diffusion coefficient. The ILs used gave no problems of miscibility with PIM-1 and the films obtained have, on qualitative analysis, the necessary properties to be used in gas separation processes. The introduction of ILs changes the properties and morphology of PIM-1, probably partially occupying the microporosities that underlie the gas separation process of this class of polymers. The change in these properties is obviously related to the concentration of the ILs, and in some cases, allows the solubility of CO_2_ in the composite to be increased. To this end, we demonstrate the positive effect of ILs in enhancing the CO_2_ solubility, as membranes with PIM-1/[BMIM][Ac] 4/1 ratio nearly doubled their CO_2_ solubility at 0.8 bar compared to PIM-1 alone, being still highly permeable. However, this was not the case for [BMIM][Succ], as its presence caused a “negative” effect on S and permeability of the blends. In general, the presence of ILs into the membrane still decreases the permeability, however an improved CO_2_ S is still possible by selecting the finest ILs and by tuning its loading. The here reported preliminary studies will allow us to optimize blended membranes and demonstrated as PIM-1/IL composite plays an important role in improving the gas solubility and probably the overall gas separation performance of PIM-1 membranes.

## Figures and Tables

**Table 1:** CO_2_ Solubility coefficients at 0.8 bar pressure derived from the direct sorption experiments.

Membrane	CO_2_ Solubility ^1^
PIM-1	0.47
PIM-1/[BMIM][Succ] 10/1	0.22
PIM-1/[BMIM][Succ] 4/1	0.33
PIM-1/[BMIM][Succ] 2/1	0.18
PIM-1/[BMIM][Ac] 10/1	0.32
PIM-1/[BMIM][Ac] 4/1	0.86
PIM-1/[BMIM][Ac] 2/1	0.69

^1^ cm^3^ (STP)/cm^3^ cmHg.

**Table 2:** CO_2_ Permeability at 1 bar pressure derived from flow cell experiments.

Membrane	CO_2_ Permeability (Barrer ^1^)
PIM-1	5177
PIM-1/[BMIM][Succ] 10/1	275
PIM-1/[BMIM][Succ] 4/1	304
PIM-1/[BMIM][Succ] 2/1	421
PIM-1/[BMIM][Ac] 10/1	2090
PIM-1/[BMIM][Ac] 4/1	171
PIM-1/[BMIM][Ac] 2/1	1053

^1^ 1 Barrer = 10^−10^ cm^3^ (STP) cm/(cm^2^ s cmHg)).

**Table 3:** Comparison of the experimental data with other literature based on PIM-based gas separation membranes.

Membrane	CO_2_ Permeability (Barrer ^1^)	CO_2_ Permeability PIM-1 Ref. (Barrer ^1^)	Ref.
PIM-1	5167 (@1 Bar, 40 °C)	5167	This work
PIM-1/[BMIM][Ac] 10/1	2090 (@1 Bar, 40 °C)	5167	This work
PIM-1/[BMIM][Ac] 2/1	1053 (@1 Bar, 40 °C)	5167	This work
IL@MOF/PIM−5 %	9420 (@4 Bar, 35 °C)	4110	[17]
PIM-1 -IL1 (pseudo-IL tetrazole-type)	1043 (@6.86 Bar, 25 °C)	7340	[30]
PIM-1/Matrimid (95:5)	3355 (@3.5 Bar, 35 °C)	3815	[13]
PIM-1/Matrimid (50:50)	155 (@3.5 Bar, 35 °C)	3815	[13]
PIM-1 + 5 wt%[C2mim][Tf2N]	6650 (@Bar ^2^, 30 °C)	7440	[14]

^1^ 1 Barrer = 10^−10^ cm^3^ (STP) cm (cm^−2^ s^−1^ cmHg^−1^); ^2^ unknown.

## Supplementary Materials

The following are available online at https://www.mdpi.com/article/10.3390/membranes12121262/s1, Table S1: Thickness and density values of PIM-1/ILs membrane, Figure S1: Thermal degradation of pure PIM-1 and PIM-1/ILs blended membranes by means of Thermo Gravimetric Analysis (TGA). (**A**) PIM-1 [BMIM][Ac] 10/1, 4/1, 2/1; (**B**) PIM-1 [BMIM][Succ] 10/1, 4/1, 2/1, Table S2: Degradation weight losses of PIM-1 and PIM-1/ILs membranes and ILs calculated by Thermo Gravimetric Analysis (TGA), Figure S2: FESEM captures of (**a**) PIM-1 membranes loaded with BMIM Ac ((**b**) 10/1, (**c**) 4/1, (**d**) 2/1) and of BMIM Succ ((**e**) 10/1, (**f**) 4/1, (**g**) 2/1).
