# Supplementary material for: Ionic Liquids-Polymer of Intrinsic Microporosity (PIMs) Blend Membranes for CO2 Separation
Source: Membranes (Basel). 2022 Dec 13;12(12):1262. doi: 10.3390/membranes12121262 (PMC9786291; doi:10.3390/membranes12121262)
Supplement: Supplementary file 1 [file membranes-12-01262-s001.zip › membranes-2062631-supplementary.pdf]

Supplementary Materials

Ionic Liquids-Polymer of Intrinsic Microporosity (PIMs) Blend Membranes for CO_2 Separation

Giuseppe Ferraro [1], Carmela Astorino [1,2], Mattia Bartoli [1], Alberto Martis [1,3], Stefania Lettieri [1,3,*], Candido Fabrizio Pirri [1,3] and Sergio Bocchini [1,3,*]

[1] Center for Sustainable Future Technologies (CSFT), Istituto Italiano di Tecnologia (IIT), Via Livorno 60, 10144 Turin, Italy
[2] Dipartimento di Chimica Generale ed Organica Applicata, Università di Torino, Corso Massimo D'Azeglio 48, 10125 Turin, Italy
[3] Department of Applied Science and Technology, Politecnico di Torino, Corso Duca Degli Abruzzi 24, 10129 Turin, Italy
* Correspondence: stefania.lettieri@polito.it (S.L.); sergio.bocchini@polito.it (S.B.)

Table S1. Thickness and density values of PIM-1/ILs membrane.

Membrane	Thickness (cm)	Density (g/cm³)
PIM-1	0.00259	1.955
PIM-1/[BMIM][Ac] 10/1	0.00274	0.979
PIM-1/[BMIM][Ac] 4/1	0.00094	1.501
PIM-1/[BMIM][Ac] 2/1	0.00138	1.287
PIM-1/[BMIM][Succ] 10/1	0.00192	1.356
PIM-1/[BMIM][Succ] 4/1	0.00165	1.479
PIM-1/[BMIM][Succ] 2/1	0.00302	1.058

Thermal degradation analysis (TGA)

The materials were characterized by TGA in order to assess degradation pattern and the thermal properties of the synthetized membranes. Pure PIM-1 membrane shows only a thermal degradation at about 528 °C (Figure S1 a-b). Instead in the composite membranes, two main degradation steps are present at 240–260 °C and 500–520 °C corresponding to the ILs and to the polymer degradation respectively. Interestingly, in both blended membranes type, the degradation temperature of the polymer decreases with the increase of the % of ILs. The detailed are shown in Table S2.

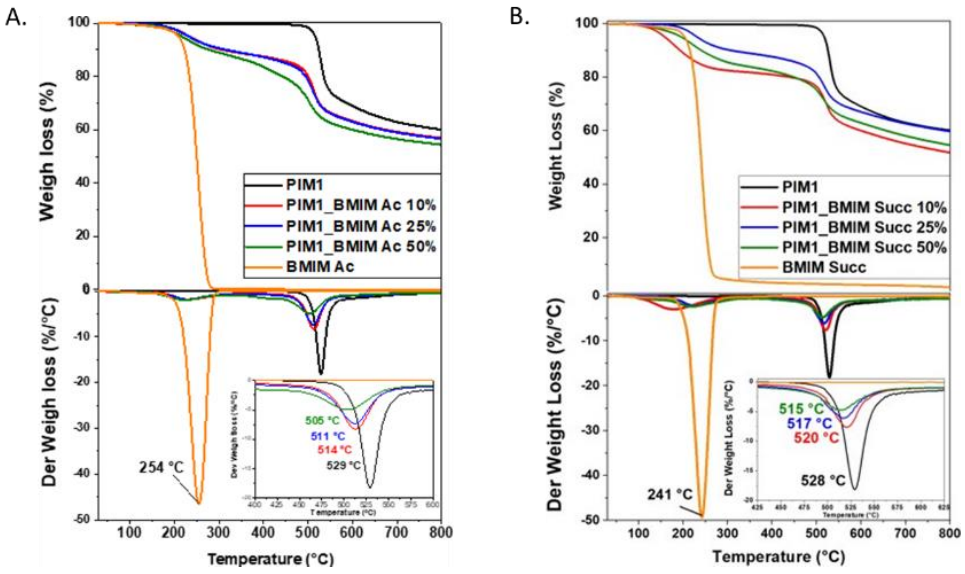

Figure S1. Thermal degradation of pure PIM-1 and PIM-1/ILs blended membranes by means of Thermo Gravimetric Analysis (TGA). (**A**) PIM-1 [BMIM][Ac] 10/1, 4/1, 2/1; (**B**) PIM-1 [BMIM][Succ] 10/1, 4/1, 2/1.

Table S2. Degradation weight losses of PIM-1 and PIM-1/ILs membranes and ILs calculated by Thermo Gravimetric Analysis (TGA).

Membrane	1st weight loss	2nd weight loss
PIM-1	528–529	-
PIM-1/[BMIM][Ac] 10/1	254	514
PIM-1/[BMIM][Ac] 4/1	254	511
PIM-1/[BMIM][Ac] 2/1	254	505
[BMIM][Ac]	254	-
PIM-1/[BMIM][Succ] 10/1	241	520
PIM-1/[BMIM][Succ] 4/1	241	517
PIM-1/[BMIM][Succ] 2/1	241	515
[BMIM][Succ]	241	-

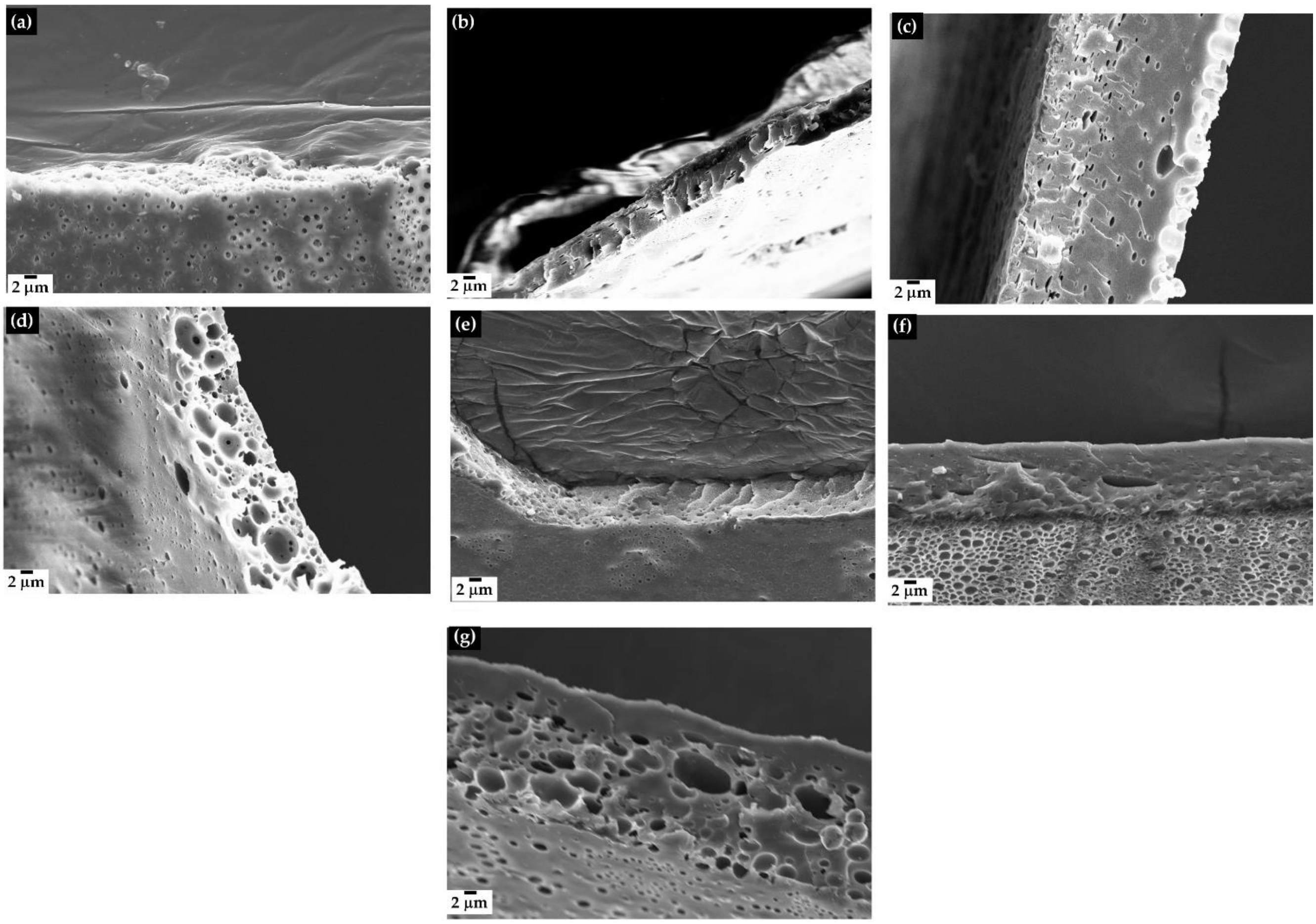

Figure S2. FESEM captures of (**a**) PIM-1 membranes loaded with BMIM Ac ((**b**)10/1, (**c**)4/1, (**d**)2/1) and of BMIM Succ ((**e**)10/1, (**f**)4/1, (**g**)2/1).

As shown in Figure S2a, PIM-1 showed a porous surface with appreciable porous network without any visible cracks showing a thick ranging from 13 up to 20 μm. As reported in Figure S2c, PIM1 cross section confirming the absence of diffuse pores. As shown in Figure S2 b and c, the addition of up to 10/1 or 4/1 of BMIM Ac loading, reduce the pore size without modifying the thickness. A similar behavior waws observed in by adding the same amount of BMIM Succ as reported in Figures S2e and S2f. The addition of both BMIM Ac and BMIM Succ (Figure S2d and S2g respectively) induced the formation of large porous network with an average size over 3μm. The pores enlargement was reasonably due to the IL filling that altered the structure of PIM-1 leaving a cavities after the cryofracturing process. The use different ILs did not affect the morphologies of the membranes that were mainly related to the amount of IL added.
